# Effects of Cigarette Smoking and 3‐Day Smoking Abstinence on Translocator Protein 18 kDa Availability: A [^18^F]FEPPA Positron Emission Tomography Study

**DOI:** 10.1111/adb.70024

**Published:** 2025-04-04

**Authors:** Arthur L. Brody, Andre Y. Sanavi, Renee Beverly‐Aylwin, Natalie Guggino, Anna K. Mischel, Alvin Wong, Ji Hye Bahn, Mark G. Myers, Brinda Rana, David Vera, Kishore K. Kotta, Jeffrey H. Meyer, Jared W. Young, Carl K. Hoh

**Affiliations:** ^1^ Department of Psychiatry University of California San Diego San Diego California USA; ^2^ Department of Research VA San Diego Healthcare System San Diego California USA; ^3^ Department of Psychiatry VA San Diego Healthcare System San Diego California USA; ^4^ Department of Radiology University of California San Diego San Diego California USA; ^5^ Azrieli Centre for Neuro‐Radiochemistry, Brain Health Imaging Centre, Campbell Family Mental Health Research Institute CAMH Toronto Canada; ^6^ Department of Psychiatry University of Toronto Toronto Canada

**Keywords:** Tobacco Dependence, Positron Emission Tomography, Neuroinflammation

## Abstract

With the many negative health consequences of cigarette smoking, quitting is known to improve health in multiple domains. Using positron emission tomography/computed tomography (PET/CT) scanning, our group previously demonstrated that smokers have lower levels than nonsmokers of translocator protein binding both acutely and following overnight abstinence. Here, we sought to determine the effects of longer smoking abstinence on this marker of gliosis for microglia and astroglia, as well as explore associations between the marker and smoking‐related symptoms. This observational study was performed in an academic VA medical centre. Fifty‐nine generally healthy Veterans who were either nonsmokers (*n* = 15) or smokers (*n* = 44) participated in the study. Participants completed an intake visit to evaluate for inclusion/exclusion criteria, [^18^F]FEPPA PET/CT scanning and a structural magnetic resonance imaging scan. Smokers were alternately assigned either to smoke to satiety (*n* = 24) before scanning or undergo three nights of continuous abstinence prior to scanning using contingency management (*n* = 20 completed this protocol and scanning). The smoker satiety group had a significantly lower mean whole brain (WB) standardized uptake value (SUV) for [^18^F]FEPPA binding than both the nonsmoking (−15.3%) and abstinent smoker (−12.3%) groups. The nonsmoking control and abstinent smoker groups had mean WB SUVs that were not significantly different from one another (3.0% group difference). In an exploratory analysis, a significant inverse relationship was found between WB SUVs and mood ratings for smokers, indicating that higher levels of TSPO binding were associated with worse mood. The central findings here support previous studies demonstrating lower levels of the marker for gliosis in satiated smokers and imply normalization with elimination of cigarette smoke constituents from the body, although other explanations for study results (e.g., alterations in radioligand delivery or clearance of radioligand by cigarette smoke constituents) are possible. These findings may represent a previously unknown health benefit of quitting smoking.

## Graphical abstract

Cigarette smoking is associated with low levels of whole brain (WB) binding of a PET radioligand for the translocator protein, a marker for gliosis. Here, we demonstrate that, compared to non‐smoking controls (group 0), this PET marker is lower in smokers who smoke to satiety prior to scanning (group 1), but is comparable between non‐smokers and smokers who maintain abstinence for 3 days (group 2). Study findings indicate that a potentially detrimental health effect of smoking resolves with short‐term abstinence.

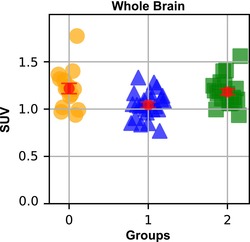

## 1 Introduction

Chronic cigarette smoking (CS) is associated with many negative health outcomes [1], including immune system–related illnesses, cancer, cardiovascular disease, lung disease, diabetes [2, 3] and impaired wound healing [4, 5]. When cigarette smokers quit and maintain abstinence, positive health changes begin to occur within minutes and the elevated risks of diseases and other detrimental effects decline over time [2, 3]. A specific effect of CS that likely contributes to some of the health consequences listed above is abnormal inflammatory function.

When functioning normally, neuroinflammation has the positive effects of tissue repair in response to central nervous system (CNS) injury, enhanced plasticity and neuroprotection [6, 7]. In contrast, abnormally low levels of neuroinflammation impair response to CNS injury and can lead to cognitive impairment [6], while abnormally high levels of neuroinflammation are associated with neurological illnesses (e.g., Alzheimer's disease, Parkinson's disease and amyotrophic lateral sclerosis) [7, 8]. Put simply, dysfunction of normal neuroinflammatory processes (either decreased or increased) is a health risk. Therefore, it is important to characterize the impact of CS on neuroinflammation, as it can have significant implications for brain health and cognitive function.

Because of the role of neuroinflammation in disease processes, much research has focused on the development of positron emission tomography (PET) radioligands for measuring markers of neuroinflammatory change. In this context, the translocator protein 18 kDa (TSPO) has been a molecule of interest because it is located in the mitochondrial membrane of microglia [7], microglia are the main resident immune cells of the CNS [9, 10, 11] and TSPO expression increases when microglia are activated [10, 12]. Furthermore, TSPO levels are elevated in conditions and illnesses in which microglia and astroglia are activated or changing toward an activated state [7, 10, 11, 13, 14, 15]. While TSPO is associated with microglial and, to a lesser extent, astroglial activation, it has incomplete cellular specificity and may be present in other cells, such as endothelial and infiltrating peripheral inflammatory cells [7, 14].

For the study presented here, ^18^F‐labelled fluoroethoxybenzyl‐*N*‐(4‐phenoxypyridin‐3‐yl) acetamide ([^18^F]FEPPA) was used with PET scanning, as it labels TSPO reliably [16] with high affinity [17, 18] and therefore has sensitivity to account for small between‐group differences [16, 17, 18, 19] and genetic TSPO predispositions [20]. Prior studies using [^18^F]FEPPA PET scanning include ones examining healthy populations [21, 22, 23], psychotic disorders [24, 25], Parkinson's disease [26, 27], major depressive disorder [28, 29] and cognitive impairment [30, 31].

Recent literature reviews of preclinical research [32, 33, 34] conclude that nicotine has more anti‐ than pro‐inflammatory effects, including overall anti‐inflammatory effects on pathways including microglia. These reviews report that nicotine binding to α7 nicotinic acetylcholine receptors (nAChRs) on microglia triggers an anti‐inflammatory cascade that alters microglial polarization and activity, cytokine release and intracellular calcium concentrations, leading to neuroprotection. However, while most studies report anti‐inflammatory properties of nicotine, evidence of increased neuroinflammation with CS exposure [35, 36] and nicotine administration [36] has also been reported. Thus, the complete pathway of nicotine's effects has not been fully elucidated, likely because neuroinflammation is a complex process resulting from multiple genes and signalling pathways.

As for studies of human CS, two prior studies by our group demonstrate that cigarette smokers have low levels of TSPO binding compared with nonsmokers. In the first of these studies [37], a second‐generation PET radioligand for TSPO ([^11^C]DAA1106) was used to examine effects of acute CS on TSPO availability. Based on the studies cited above, we had hypothesized that smokers would have abnormally low binding of [^11^C]DAA1106 on PET scanning, indicating less gliosis and that effects would occur globally throughout the brain, since prior research demonstrates widespread effects of CS when studying systems (e.g., the nAChR system) distributed throughout the brain [38, 39, 40, 41, 42]. The central finding of this study was that the PET outcome measure (whole brain [WB] standardized uptake value [SUV]) was significantly lower (mean—16.8%) in smokers who smoked to satiety immediately before scanning than in nonsmokers. Consistent with this global finding, analysis of smaller subcortical volumes of interest (VOIs) revealed a that all VOIs had a significant between‐group effect, due to smokers having lower SUVs than nonsmokers (14.6% to 19.7%). In the second study [43], we examined smokers who maintained abstinence overnight and the effect of this abstinence on TSPO availability. Smokers and nonsmokers underwent the same procedures as in the preceding study, with the exception being that smokers initiated abstinence prior to midnight on the night before the [^11^C]DAA1106 PET session (verified by an exhaled carbon monoxide [CO] level ≤ 4 ppm). The central study finding was that overnight abstinent smokers had significantly lower mean WB SUVs (by roughly 16.3%) than nonsmokers. Also, smaller VOIs had significant between‐group effects (*p* values < 0.0005 to 0.03), with abstinent smokers having lower SUVs in all VOIs. Taken together, these studies indicate that gliosis is reduced with acute smoking and this reduction persists through overnight abstinence.

Here, we sought to determine if a longer period of smoking abstinence would result in normal levels of TSPO binding. Because nicotine, a primary addictive constituent of tobacco smoke, has a half‐life of about 2–3 h [44] and its major metabolite (cotinine) [45, 46] and the combination of all its major metabolites [47] have half‐lives of approximately 16–19 h, we hypothesized that smokers abstinent for 3 days would have levels of the PET marker for gliosis similar to nonsmokers. In addition, because prior research found associations between the PET marker for gliosis and smoking withdrawal symptoms [37] (and depression [48]), we conducted an exploratory analysis examining associations between the marker for gliosis and withdrawal symptoms at the time of PET/CT scanning.

## 2 Methods

**FIGURE 1:**
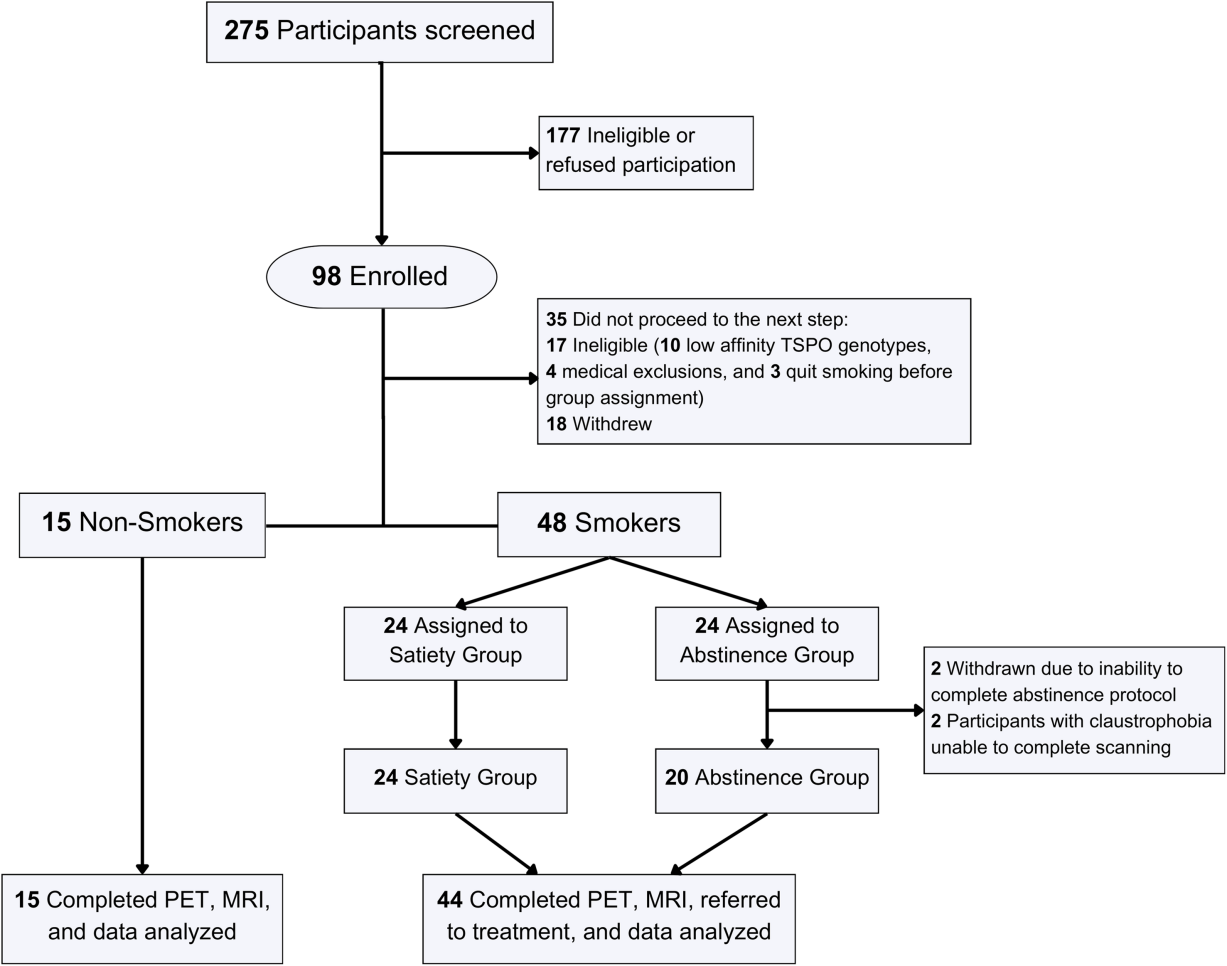
Consort diagram showing flow of participants through the study.

Fifty‐nine generally healthy adult Veterans (*n* = 15 nonsmokers and *n* = 44 smokers) completed the study and had usable data. Participants underwent the following study procedures (described in more detail below): telephone screening, in‐person intake visit, a satiety or 3‐day smoking abstinence protocol prior to PET/computed tomography (CT) scanning for participants who were smokers (alternate assignment), an [^18^F]FEPPA PET/CT scan, a structural magnetic resonance imaging (MRI) scan and (for smokers) referral to smoking cessation treatment. The study was approved by the IRB at the VA San Diego Healthcare System (#1204452). An additional four cigarette smokers were enrolled and assigned to the abstinence protocol, but two did not complete it and two started PET/CT scanning but were unable to complete it due to claustrophobia (Figure 1).

At the intake visit, participants were evaluated to determine if they met inclusion/exclusion criteria. Inclusion criteria were (1) adult Veterans (18–75 years old) who were daily cigarette smokers meeting criteria for tobacco use disorder [49] and interested in attempting to quit or nonsmokers (never users or > 1 year tobacco free); (2) ability to read, write and provide informed consent; and (3) smoking status confirmed by exhaled CO levels (≥ 8 ppm for smokers, < 8 ppm for nonsmokers). Exclusion criteria were (1) any acute psychiatric diagnosis (including mood, anxiety, psychotic and substance use disorders) within the past year; occasional drug/alcohol use not meeting criteria for abuse/dependence was not exclusionary, but participants were instructed to abstain from use for > 48 h prior to PET/CT scanning; (2) history of conditions that could affect the CNS at the time of scanning (e.g., history of severe head trauma, epilepsy, or other neurological diseases); (3) daily use of anti‐inflammatory medications; (4) unstable cardiovascular, liver or renal disease, which might make tolerating procedures difficult; (5) low affinity TSPO genotype; and (6) pregnancy, due to the theoretical risk of radiation exposure to the foetus.

During the intake visit, medical records were reviewed, and rating scales were administered to confirm eligibility and collect basic background and symptom information. Rating scales included the smoker's profile form [38] (demographic, race/ethnicity, educational level, medications, smoking history and other information) and Fagerström Test for Nicotine Dependence [50, 51] (FTND; severity of nicotine dependence). Because prior research demonstrates that genotyping can determine an individual's TSPO affinity subtype (high, medium or low), and these affinities affect binding for radioligands determining TSPO availability [20, 52], a saliva sample was also collected at the intake visit for genotyping of rs6971 within the TSPO gene. Using this sample, DNA was extracted from buccal cells using the Oragene OG‐500 kit (DNA Genotek; Ottawa, Ontario, Canada) according to manufacturer protocol. Genotyping of rs6971 within the TSPO gene was performed by polymerase chain amplification (Forward Primer: 5′‐AAGCGTGACGGCCACCACATCA‐3′; Reverse Primer: 5′‐CCTGACTCCCAAATCCAGTG‐3′) of a 362 base pair fragment of the *TSPO* gene containing the rs6917 followed by restriction enzyme digestion with *NruI* (New England Biolabs; Ipswich, MA) in the laboratory of a study co‐investigator (B.R.). Only participants with the high or medium affinity genotypes (> 90% of North Americans [22]) were included in PET/CT data analysis to avoid a potential confound.

After the intake visit, participants who were smokers were alternately assigned to either the satiety group or the abstinence group. Smokers assigned to the satiety condition were instructed to smoke as per their usual habit until the day of PET/CT scanning. Smokers assigned to the abstinence condition were instructed to stop smoking or using any nicotine containing products three nights prior to PET/CT scanning. Participants assigned to this condition met with study personnel and had abstinence checks 2 days, 1 day and the day of PET/CT scanning where they were required to report continuous abstinence from smoking and use of nicotine‐containing products and have exhaled CO levels ≤ 8 ppm for the 2 days prior visit and ≤ 4 ppm for the day before and day of PET/CT scanning visits in order to continue to scanning. Contingency management (CM) was used to encourage smoking abstinence, with the following protocol: $20 for completing one night of abstinence, an additional $30 for two nights of abstinence and an additional $120 for all three nights of abstinence. Participants in the CM group were told whether or not they would be receiving the extra payments at each abstinence check visit. All participants received compensation for study assessments and were paid by check or bank transfer after study completion.

On the PET/CT scanning day, participants arrived at the VA San Diego PET/CT Center at midday. State rating scales were obtained, including the Minnesota Nicotine Withdrawal Scale (MNWS) [53] and analogue scales for craving, mood and anxiety. The MNWS‐revised comprehensively assessed cigarette withdrawal symptoms (including irritability, anxiety, depression and craving). Analogue ratings of craving, mood and anxiety were single items ranked from 1 to 7 (none to extremely strong [craving and anxiety] or very bad to very good [mood]). Following rating scale administration, both a breathalyser (AlcoMatePro) and urine toxicology screen (Test Country I‐Cup Urine Toxicology Kit) were obtained. Given that roughly 18% of American adults use marijuana [54], CS and marijuana use are highly comorbid [55] and urine toxicology screens may remain positive for 3–7 days for marijuana with a single use [56], participants were not excluded for a positive urine toxicology screen for marijuana or other recreational drugs, but were instructed to abstain for at least 48 h prior to PET/CT scanning and verbally confirmed this point at the testing session. Height and body weight were obtained for all all participants, and a urine pregnancy test (β‐HCG) (Test Country Cassette Urine Pregnancy Test) was obtained for female participants of childbearing potential were also obtained.

Following the above tests (which took approximately 1–2 h to complete), participants had an intravenous line placed by a nuclear medicine technician in a room adjacent to the PET/CT scanner. Participants who were smokers in the satiety group were taken to an outdoor area 10 min prior to scanning and smoked to satiety (18 of 24 smokers smoked one cigarette [range 0.75 to 3 cigarettes]), while smokers in the abstinence group and nonsmokers did not smoke prior to scanning. Immediately prior to PET/CT scanning (after smoking to satiety for the smoker satiety group), an exhaled CO level was measured with the Micro+ Smokerlyzer Breath CO Monitor (Bedfont Scientific, Ltd, UK), with CO levels of ≥ 8 ppm considered to be consistent with recent smoking and levels ≤ 4 ppm considered consistent with nonsmoking status. Participants were then positioned on the PET/CT scanner and received a bolus injection of ~185 MBq of [^18^F]FEPPA, followed by a dynamic PET scan of the brain for 90 min. PET/CT scans were obtained on a Siemens Biograph mCT Flow PET/CT scanner (Siemens Healthcare, Erlangen, Germany) in 3D mode. Scans contained 64 transaxial slices with a 500‐mm field of view and a 5‐mm in‐plane spatial resolution (FWHM). Study investigators obtained an investigational new drug approval from the FDA (IND 141607) for the use of [^18^F]FEPPA here, and it was produced using an established method [57, 58]. Following the PET/CT scan, state rating scales (MNWS and analogue craving, anxiety and mood) were again administered.

A structural MRI scan of the brain was obtained within a few weeks of PET/CT scanning and co‐registered with the PET/CT scans to aid in localization of anatomical regions. Structural MRI scans were obtained on a Siemens 3T Skyra Fit using a 3D T1 MPRAGE sequence with FOV = 220 mm, TR = 2300, TE = 2.99 ms, flip angle = 9°, slice thickness = 1.2 and scan time = 5:06. Total MRI scanning time was < 20 min.

Similar to previous research by our group [37, 38, 39, 43, 59, 60, 61, 62], PET‐to‐MRI co‐registration was performed using the PMOD quantitative parametric mapping software (Bruker, U.S.), and automated VOIs were determined on MRI and transferred to co‐registered PET/CT scans. The primary VOI was WB determined with FSL FAST for reasons cited above. Since regional differences were possible, VOIs were also determined for the amygdala, caudate, globus pallidus, hippocampus, nucleus accumbens, putamen and thalamus, similar to prior research [63, 64].

To obtain a quantitative measurement of VOI binding of TSPO in brain, SUVs were the primary outcome measure and were calculated using the standard definition: SUV = mean tissue activity concentration (Bq/mL)/(injected dose [Bq]/body weight [g]). Mean tissue activity concentration from 18.5 to 80 min post‐injection was used, based on time activity curves from this line of research demonstrating relatively stable radioligand levels during this period. SUV was used as the primary outcome measure because it avoids invasive arterial blood sampling and this method was not feasible at our facility. It is also noted that SUV has been shown to strongly correlate with total volume of distribution (Vt) values [65, 66], has good test–retest reproducibility [66] and has less intersubject variability than that for Vt [65] in studies using similar radioligands. Following the PET/CT session, smokers were referred to a standard 12‐week smoking cessation program at the VA San Diego Healthcare System.

Statistical analyses were performed on PET/CT outcomes to test study hypotheses using the SPSS version 29.0 (SPSS Inc., Chicago, IL). For baseline variables, groups were compared with chi‐square (for categorical variables) and *t*‐tests (for continuous variables). For PET/CT data, study groups were compared with an overall analysis of variance (ANOVA), with WB SUV as the dependent variable, group (nonsmoker, satiety smoker and abstinent smoker) as the between‐subject factor, and genotype as a nuisance covariate. For completeness, statistical analyses with the same structure were performed for the smaller subcortical VOIs. In addition to the primary study analysis, an exploratory analysis was performed including all smokers to determine partial correlations (controlling for TSPO genotype and smoking group [satiety or abstinence]) between PET/CT WB SUVs and mean values from the MNWS and craving, mood and anxiety scales. Alpha was set to *p <* 0.05.

## 3 Results

Study groups were well‐matched for baseline variables (Table 1) with no significant differences in age, sex, race, ethnicity, education (years), height, weight, body mass index (BMI), alcohol drinks per week, or TSPO affinity genotype (*p* = 0.30–0.98). The two smoker groups did not differ at baseline in number of cigarettes smoked per day, pack‐year smoking history, percentage of menthol cigarette smokers or FTND scores (*p* = 0.60–0.90).

**TABLE 1:** Demographic, health, and smoking‐related variables for the study groups.

Variable	Nonsmokers (*n* = 15)	Smoker satiety (*n* = 24)	Smoker abstinent (*n* = 20)
Demographic			
Age	55.8 (± 13.4)	54.2 (± 11.2)	54.6 (± 12.4)
Sex (% female)	26.7	16.7	20.0
Race (%)			
Asian/PI	13.3	16.7	20.0
Black	26.7	25.0	10.0
White	53.3	50.0	60.0
> 1 or other	0	8.3	10.0
Ethnicity (%Hispanic)	0	8.3	10.0
Education (years)	14.9 (± 2.5)	14.6 (± 2.4)	14.2 (± 1.8)
Health‐related			
Height (centimetres)	171.5 (± 8.7)	172.6 (± 8.8)	175.5 (± 7.5)
Weight (kilograms)	86.1 (± 13.8)	85.3 (± 16.6)	91.2 (± 17.7)
Body mass index	29.3 (± 4.7)	28.7 (± 5.8)	29.5 (± 5.1)
Alcohol drinks/week	2.0 (± 3.8)	2.7 (± 4.1)	3.5 (± 6.0)
TSPO (% high affinity genotype)	66.7	66.7	70.0
Smoking‐related (baseline)			
Cigarettes per day	n/a	16.5 (± 7.4)	16.1 (± 7.5)
Pack‐year smoking history	n/a	28.7 (± 21.4)	30.3 (± 24.5)
Menthol cigarette smokers (%)	n/a	37.5	30.0
FTND	n/a	4.2 (± 2.7)	4.3 (± 1.8)
Smoking‐related (PET/CT scan)			
MNWS	n/a	15.2 (± 9.7)	15.0 (± 9.0)
Craving (analogue)	n/a	3.6 (± 1.6)	3.8 (± 1.8)
Anxiety (analogue)	n/a	2.6 (± 1.3)	2.7 (± 1.1)
Mood (analogue)	n/a	4.7 (± 1.6)	4.9 (± 1.3)
Exhaled CO (ppm)	1.9 (± 0.7)	17.0 (± 10.1)***	2.1 (± 1.0)

*Note:* All values are presented as means (± standard deviations) or percentages. Using analyses of variance and Student *t*‐tests for continuous variables and *χ* ^2^ tests for categorical variables, no between‐group tests were significant, except for the comparison of exhaled CO between the smoker satiety group and both of the other study groups where ***p < 0.0001. Abbreviations: CO, carbon monoxide; FTND, Fagerström Test for Nicotine Dependence; MNWS, Minnesota Nicotine Withdrawal Scale; PI, Pacific Islander; ppm, parts per million; TSPO, translocator protein.

At the PET/CT scanning visit, all alcohol breathalyser and urine pregnancy tests were negative. All participants reported no drug use (including marijuana) for 48 h prior to PET/CT scanning; however, a small number of participants had urine toxicology screens that were positive for THC (*n* = 0, 3 and 2 for the nonsmoker, satiety smoker and abstinent smoker groups, respectively). Smoking symptom rating scales obtained at the PET/CT session were not significantly different between the two smoker groups (Student *t*‐tests, *p* = 0.63–0.93 for the MNWS and analogue rating scales; Table 1), which was likely due to scales being administered prior to and after the PET/CT scan (roughly 1 and 3 h after arriving for the study visit), such that both groups had moderate withdrawal symptoms. As expected, the smoker satiety group had a higher exhaled CO immediately prior to scanning than the other study groups (Student *t*‐tests, p's < 0.0001). The injected radioligand dose was similar for the nonsmoker, satiety smoker and abstinent smoker groups (196.4 ± 5.0, 195.9 ± 5.8 and 195.1 ± 5.0 MBq, respectively; *p* = 0.72).

**FIGURE 2:**
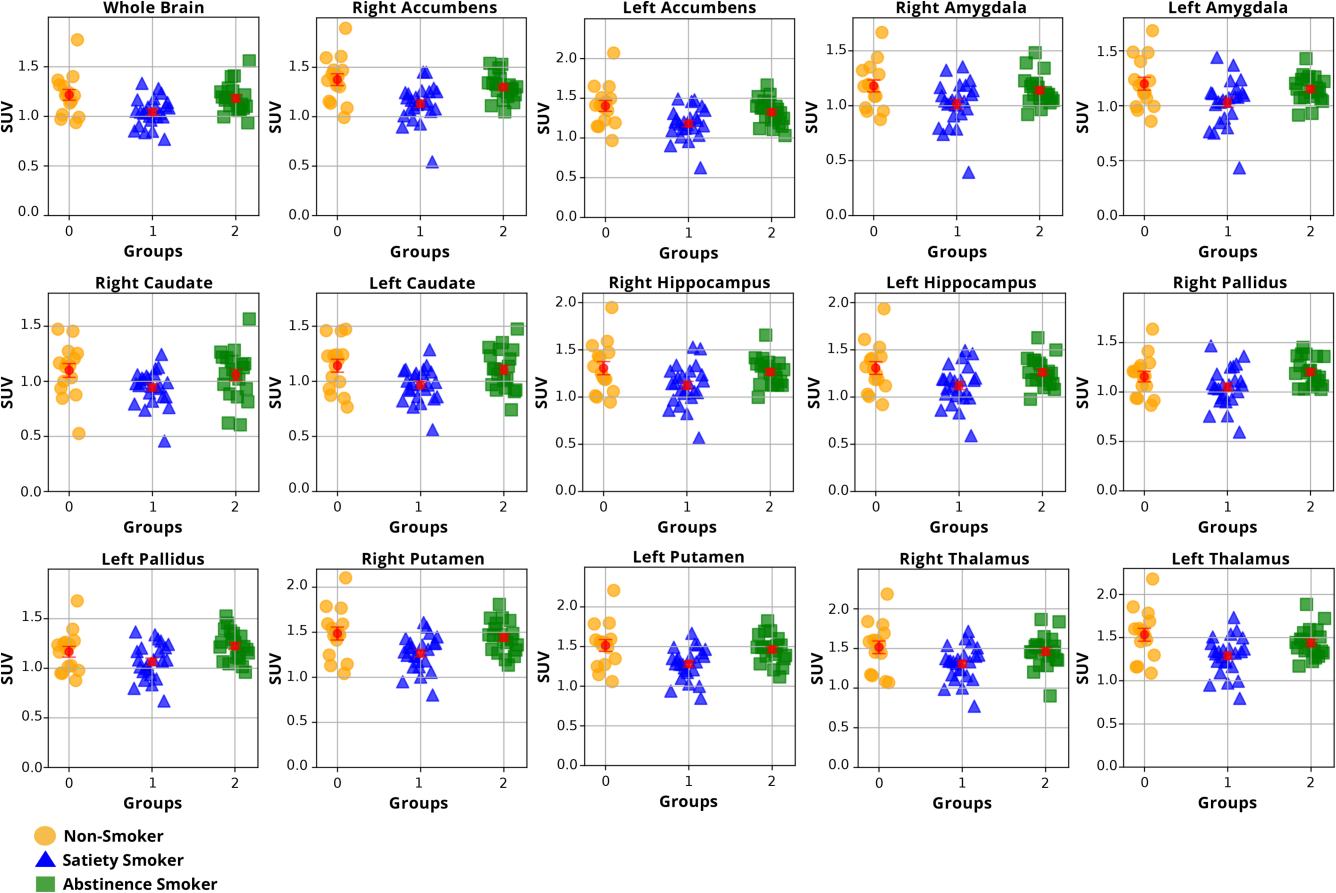
Scatterplots showing standardized uptake values (SUVs) for the whole brain (WB) and smaller volumes of interest for the three study groups (nonsmokers, smokers in the satiated state and smokers in 3 days of continuous abstinence state).

The primary overall analysis of PET/CT data revealed a significant main effect of group (*F*_[2, 55]_ = 6.3, *p* = 0.003), due to the smoker satiety group having significantly lower WB SUVs than both the nonsmoking control (−15.3%; *F*_[1, 36]_ = 9.4, *p* = 0.004) and abstinent smoker (−12.3%; *F*_[1, 41]_ = 9.4, *p* = 0.004) groups (Table 2 and Figure 2). The nonsmoking control and abstinent smoker groups had mean WB SUVs that were similar to one another (3.0% group difference; *F*_[1, 32]_ = 0.4, *p* = 0.51). For the smaller VOIs, 13 of the 14 SUVs had a significant main effect of group (*F*_[2, 55]_ = 4.0–9.1, *p* ≤ 0.001–0.02; Table 2), while the remaining VOI approached significance (*F*_[2, 55]_ = 2.9, *p* = 0.06). These results were due to the smoker satiety group having lower SUVs than both the nonsmoking control (range: 8.9%–19.4%) and smoking abstinence (range: 10.9%–13.8%) groups. As an aside, since body weight was nonsignificantly higher in the abstinent smoker group (Table 1) and was used to calculate SUV, an analysis was run using the same variables as above with the addition of body weight as a nuisance covariate; in this analysis, the significant main effect of group was essentially unchanged (*F*_[2, 54]_ = 6.7, *p* = 0.002).

**TABLE 2:** Standardized uptake values (SUVs) for the whole brain and smaller volumes of interest.

Brain region		Nonsmoker group (*n* = 15)	Smoker satiety group (*n* = 24)	Smoker abstinence group (*n* = 20)	*p*
Whole brain		1.22 (± 0.21)	1.04 (± 0.14)	1.18 (± 0.15)	0.003
Accumbens	R	1.37 (± 0.23)	1.13 (± 0.19)	1.30 (± 0.13)	<0.001
	L	1.40 (± 0.27)	1.18 (± 0.20)	1.32 (± 0.16)	0.005
Amygdala	R	1.18 (± 0.21)	1.01 (± 0.21)	1.14 (± 0.14)	0.02
	L	1.20 (± 0.23)	1.03 (± 0.21)	1.15 (± 0.12)	0.02
Caudate	R	1.10 (± 0.24)	0.94 (± 0.16)	1.06 (± 0.23)	0.06
	L	1.14 (± 0.23)	0.97 (± 0.15)	1.11 (± 0.18)	0.01
Globus Pallidus	R	1.16 (± 0.21)	1.05 (± 0.20)	1.20 (± 0.14)	0.02
	L	1.17 (± 0.21)	1.07 (± 0.18)	1.22 (± 0.15)	0.02
Hippocampus	R	1.31 (± 0.27)	1.12 (± 0.21)	1.27 (± 0.15)	0.02
	L	1.31 (± 0.26)	1.12 (± 0.21)	1.26 (± 0.15)	0.02
Putamen	R	1.49 (± 0.28)	1.27 (± 0.19)	1.44 (± 0.17)	0.004
	L	1.51 (± 0.29)	1.28 (± 0.20)	1.46 (± 0.18)	0.003
Thalamus	R	1.51 (± 0.31)	1.31 (± 0.22)	1.46 (± 0.21)	0.02
	L	1.53 (± 0.29)	1.29 (± 0.22)	1.44 (± 0.17)	0.005

*Note*: All values are mean ± standard deviation. L, left; R, right. Each region was analysed using an analysis of variance, with group as a between‐subject factor and genotype as a nuisance covariate. *p* values are for main effect of group. Automated regions listed here were generated using the FSL toolkit.

**FIGURE 3:**
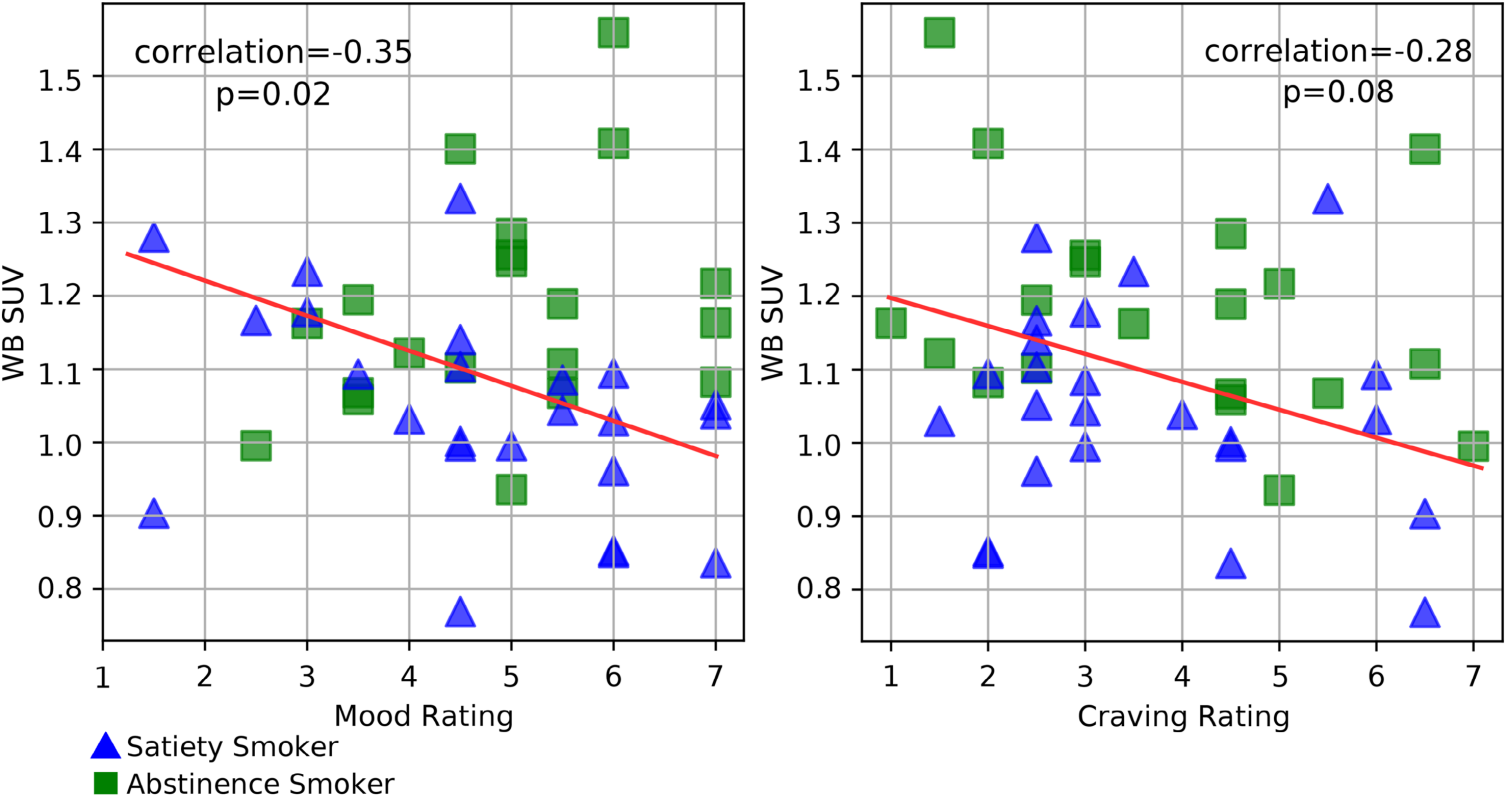
Scatterplots showing exploratory correlations between WB SUV and cigarette withdrawal ratings for mood and craving at the time of PET/CT scanning.

In the exploratory analyses of symptom rating scales at the time of PET/CT scanning, a significant inverse relationship was found between WB SUV and mood ratings for the whole smoker group (partial correlation coefficient = −0.35, *p* = 0.02; Figure 3), while a trend toward a significant inverse relationship was found between WB SUV and the single item analogue craving rating for the whole smoker group (partial correlation coefficient = −0.28, *p* = 0.08). No other correlations between WB SUV and symptom ratings were significant (*p* values ranged from 0.27 to 0.38).

## 4 Discussion

The main study results were that smokers who smoked to satiety had lower levels of the marker for gliosis than nonsmokers and this effect was not present in smokers who maintained CS abstinence for 3 days. Using a different PET/CT radiotracer and an independent group of participants, this study strengthens support for previous findings of decreased TSPO binding with acute smoking [37] and is consistent with the finding of decreased binding with overnight abstinence [43]. Furthermore, the magnitude of the low binding here (−15.3%) was similar to that found in the previous studies (−16.8 and roughly −16.3%, respectively) and also similar to findings in chronic alcohol use disorder [67, 68]. These findings in the brain with acute CS are consistent with known effects of smoking in the body, namely, impaired inflammation and prolonged wound healing [4, 5].

The new finding that smokers abstinent for 3 days did not have decreased TSPO binding compared with nonsmokers suggests that lowered levels of gliosis from smoking resolve during the period between overnight and 3 days of smoking abstinence. As noted above, inhaled cigarette smoke has thousands of constituents [2, 69, 70] with a range of half‐lives. Nicotine, a primary addictive constituent of tobacco smoke, has a half‐life of about 2–3 h [44], while its major metabolite (cotinine) [45, 46] and the combination of all its major metabolites [47] have half‐lives of approximately 16–19 h. Other constituents of tobacco smoke (e.g., benzene, CO) have half‐lives ranging from 1 to 4.6 h [71, 72]. Therefore, PET/CT results from 3 days of smoking abstinence may be explained by elimination of CS constituents leading to normalization of gliosis, although other explanations are possible. For example, stopping smoking may result in alterations in radioligand delivery, change in radioligand clearance associated with removal of cigarette smoke constituents or lessening of elevated cholesterol levels [73], which has previously been shown to inversely correlate with TSPO radioligand binding [68]. It should also be noted that we did not find an association between plasma nicotine levels and WB SUV in our previous study [37], which may indicate that elimination of other CS constituents is responsible for the main study result here. As a possible example, CO is a constituent of tobacco smoke that has greater affinity for haemoglobin than oxygen and negatively affects oxygen delivery to tissue, which is known to be essential for wound healing in the body [74]; therefore, it may be inferred that elimination of CO from the body during short‐term abstinence is a contributor to the main finding here. Another possible interpretation of study findings is that removal of the anti‐inflammatory effect of CS/nicotine may lead to worse withdrawal symptoms (as implied by the exploratory inverse correlation found here between WB SUV and mood). These findings also point to the need to control for CS in brain imaging studies of populations that have high rates of CS, such as those with mental illness and/or substance abuse [75].

Normalization of the marker for gliosis implies an important health improvement in smokers who quit, namely improved ability to respond to CNS injury and decreased likelihood of cognitive impairment [6]. This finding potentially puts normalization of gliosis in the same category of health benefits of quitting smoking as other short‐term benefits (e.g., drop in heart rate/blood pressure, removal of CO from the body and improvements in taste/smell and lung mucus clearance) [76].

While exploratory in nature, the inverse associations between TSPO binding and symptoms are consistent with prior research. For the mood state scale, higher TSPO binding was associated with worse mood at the time of scanning in the whole smoker group. This result is consistent with much literature demonstrating increased TSPO binding in people with major depressive disorder (MDD) [48, 77]. Higher regional TSPO binding has also been found to correlate with both greater MDD symptom severity [78] and reductions in depressive symptoms with treatment [29], although it is recognized that mood in people with MDD and mood state related to smoking may have different biological substrates. For the craving scale, greater craving had a trend toward an association with lower levels of TSPO binding, which is consistent with our previous report of greater withdrawal stimulation being associated with lower TSPO availability [37]. While correlations are consistent with prior research, results should be interpreted with caution, as we did not have specific hypotheses about these relationships and multiple correlations were run which would not have passed strict multiple comparison correction.

Limitations of the study were primarily related to the complex nature of CS and the PET/CT method used. Because CS contains thousands of constituents [79, 80], it is not possible to know which (if any) resulted in the lower levels of radioligand binding found here in smokers who smoked to satiety. In our prior study [37], we found an inverse association between cigarettes smoked per day and a PET marker for gliosis but did not find associations between the PET marker and plasma nicotine/cotinine. Because of modest sample sizes, it was not possible to definitively conclude that nicotine does not cause decreased gliosis. Given the growing prevalence of vaping [81, 82, 83, 84], which typically delivers nicotine, future research of this type could focus on people who vape to help determine the role of nicotine in the findings in this study. Additionally, building upon initial studies [85], longitudinal research could be conducted using animals to determine CS and vaping effects on TSPO binding over time. As for the PET/CT method, while radioligand binding to TSPO is thought to be substantially related to its expression during microglial and to a lesser extent, astroglial activation, TSPO is not fully selective [14, 48] and such binding may reflect inflammatory cell density [86]. In addition, the absence of arterial blood sampling precluded determination of Vt, which is a gold standard outcome measure for this type of research. Vt may control for potential confounds of between‐subject differences in radioligand delivery and binding to plasma protein [87, 88]. Finally, the cross‐sectional nature of the study did not allow for a definitive determination of normalization of TSPO levels, since participants were not scanned repeatedly over time, related to concerns about feasibility and tolerability.

Despite these limitations, several aspects of the study lend confidence to the findings. First, this study represents the third time (using independent groups of participants and two different radioligands) that acute CS (or brief abstinence) was found to result in lower TSPO binding. Second, results are consistent with the known effects of smoking in the body, including impaired inflammation and prolonged wound healing. Third, study groups were well‐matched on all variables obtained. And fourth, the time frame for resolution of lower TSPO binding was consistent with the known clearance of constituents of tobacco smoke from the body. Future research could focus on establishing which constituents of cigarette smoke alter levels of the marker for gliosis. Taken together, these findings may demonstrate a previously unknown health benefit of quitting smoking.

## Author Contributions

A.L.B., M.G.M., B.R., D.V., J.H.M., J.W.Y., and C.K.H. designed the study. A.L.B., A.Y.S., R.B.A., N.G., A.K.M., A.W., J.H.B., M.G.M., K.K.K., and C.K.H. acquired the data. A.L.B., A.Y.S., R.B.A., J.H.M., and C.K.H. analyzed and interpreted the data. All authors contributed to the writing and gave final approval for the manuscript.

## Ethics Statement

The study was approved by the IRB at the VA San Diego Healthcare System (#1204452).

## Conflicts of Interest

The authors declare no conflicts of interest.

## Data Availability

The data that support the findings of this study are available from the corresponding author upon reasonable request.

## References

[1] X. Dai , G. F. Gil , M. B. Reitsma , et al., “Health Effects Associated With Smoking: A Burden of Proof Study,” Nature Medicine 28, no. 10 (2022): 2045–2055, 10.1038/s41591-022-01978-x. PMC9556318 36216941

[2] US Department of Health and Human Services , “The Health Consequences of Smoking—50 Years of Progress. A Report of the Surgeon General,” U.S. Department of Health and Human Services, Centers for Disease Control, National Center for Chronic Disease Prevention and Health Promotion, Office on Smoking and Health, (2014).

[3] “How Tobacco Smoke Causes Disease: The Biology and Behavioral Basis for Smoking‐Attributable Disease: A Report of the Surgeon General,” (Atlanta, GA: U.S. Department of Health and Human Services, Centers for Disease Control and Prevention, National Center for Chronic Disease Prevention and Health Promotion, Office on Smoking and Health, 2010). 21452462

[4] J. Towler , “Cigarette Smoking and Its Effects on Wound Healing,” Journal of Wound Care 9, no. 3 (2000): 100–104, 10.12968/jowc.2000.9.3.25962. 11933289

[5] R. B. Goncalves , R. D. Coletta , K. G. Silverio , et al., “Impact of Smoking on Inflammation: Overview of Molecular Mechanisms,” Inflammation Research 60, no. 5 (2011): 409–424, 10.1007/s00011-011-0308-7. 21298317

[6] D. J. DiSabato , N. Quan , and J. P. Godbout , “Neuroinflammation: The Devil Is in the Details,” Journal of Neurochemistry 139, no. 2 (2016): 136–153, 10.1111/jnc.13607. 26990767 PMC5025335

[7] S. Salerno , M. Viviano , E. Baglini , et al., “TSPO Radioligands for Neuroinflammation: An Overview,” Molecules 29, no. 17 (2024): 4212, 10.3390/molecules29174212. 39275061 PMC11397380

[8] W. Zhang , D. Xiao , Q. Mao , and H. Xia , “Role of Neuroinflammation in Neurodegeneration Development,” Signal Transduction and Targeted Therapy 8, no. 1 (2023): 267, 10.1038/s41392-023-01486-5. 37433768 PMC10336149

[9] A. Waisman , F. Ginhoux , M. Greter , and J. Bruttger , “Homeostasis of Microglia in the Adult Brain: Review of Novel Microglia Depletion Systems,” Trends in Immunology 36, no. 10 (2015): 625–636, 10.1016/j.it.2015.08.005. 26431940

[10] J. Ottoy , L. De Picker , and M. S. Kang , “Microglial Positron Emission Tomography Imaging in Vivo : Positron Emission Tomography Radioligands: Utility in Research and Clinical Practice,” Advances in Neurobiology 37 (2024): 579–589, 10.1007/978-3-031-55529-9_32. 39207714

[11] N. R. Raval , R. R. Wetherill , C. E. Wiers , J. G. Dubroff , and A. T. Hillmer , “Positron Emission Tomography of Neuroimmune Responses in Humans: Insights and Intricacies,” Seminars in Nuclear Medicine 53, no. 2 (2023): 213–229, 10.1053/j.semnuclmed.2022.08.008. 36270830 PMC11261531

[12] D. C. Anthony and F. J. Pitossi , “Special Issue Commentary: The Changing Face of Inflammation in the Brain,” Molecular and Cellular Neurosciences 53 (2013): 1–5, 10.1016/j.mcn.2012.11.005. 23147112

[13] B. C. Uzuegbunam , C. Rummel , D. Librizzi , C. Culmsee , and B. Hooshyar Yousefi , “Radiotracers for Imaging of Inflammatory Biomarkers TSPO and COX‐2 in the Brain and in the Periphery,” International Journal of Molecular Sciences 24, no. 24 (2023): 17419, 10.3390/ijms242417419. 38139248 PMC10743508

[14] T. R. Guilarte , A. N. Rodichkin , J. L. McGlothan , A. M. Acanda De La Rocha , and D. J. Azzam , “Imaging Neuroinflammation With TSPO: A new Perspective on the Cellular Sources and Subcellular Localization,” Pharmacology & Therapeutics 234 (2022): 108048, 10.1016/j.pharmthera.2021.108048. 34848203 PMC9107500

[15] T. G. Luu and H. K. Kim , “18F‐Radiolabeled Translocator Protein (TSPO) PET Tracers: Recent Development of TSPO Radioligands and Their Application to PET Study,” Pharmaceutics 14, no. 11 (2022): 2545, 10.3390/pharmaceutics14112545. 36432736 PMC9697781

[16] L. Zhang , K. Hu , T. Shao , et al., “Recent Developments on PET Radiotracers for TSPO and Their Applications in Neuroimaging,” Acta Pharmaceutica Sinica B 11, no. 2 (2021): 373–393, 10.1016/j.apsb.2020.08.006. 33643818 PMC7893127

[17] S. Venneti , G. Wang , J. Nguyen , and C. A. Wiley , “The Positron Emission Tomography Ligand DAA1106 Binds With High Affinity to Activated Microglia in Human Neurological Disorders,” Journal of Neuropathology and Experimental Neurology 67, no. 10 (2008): 1001–1010, 10.1097/NEN.0b013e318188b204. 18800007 PMC2669281

[18] F. Chauveau , H. Boutin , N. Van Camp , F. Dolle , and B. Tavitian , “Nuclear Imaging of Neuroinflammation: A Comprehensive Review of [11C]PK11195 Challengers,” European Journal of Nuclear Medicine and Molecular Imaging 35, no. 12 (2008): 2304–2319, 10.1007/s00259-008-0908-9. 18828015

[19] S. Chaki , T. Funakoshi , R. Yoshikawa , et al., “Binding Characteristics of [3H]DAA1106, a Novel and Selective Ligand for Peripheral Benzodiazepine Receptors,” European Journal of Pharmacology 371, no. 2–3 (1999): 197–204. 10357257 10.1016/s0014-2999(99)00118-1

[20] D. R. Owen , R. N. Gunn , E. A. Rabiner , et al., “Mixed‐Affinity Binding in Humans With 18‐kDa Translocator Protein Ligands,” Journal of Nuclear Medicine 52, no. 1 (2011): 24–32, 10.2967/jnumed.110.079459. 21149489 PMC3161826

[21] R. Mizrahi , P. M. Rusjan , I. Vitcu , et al., “Whole Body Biodistribution and Radiation Dosimetry in Humans of a New PET Ligand, [(18)F]‐FEPPA, to Image Translocator Protein (18 kDa),” Molecular Imaging and Biology 15, no. 3 (2013): 353–359, 10.1007/s11307-012-0589-4. 22895910

[22] R. Mizrahi , P. M. Rusjan , J. Kennedy , et al., “Translocator Protein (18 kDa) Polymorphism (rs6971) Explains in‐Vivo Brain Binding Affinity of the PET Radioligand [(18)F]‐FEPPA,” Journal of Cerebral Blood Flow and Metabolism 32, no. 6 (2012): 968–972, 10.1038/jcbfm.2012.46. 22472607 PMC3367231

[23] P. M. Rusjan , A. A. Wilson , P. M. Bloomfield , et al., “Quantitation of Translocator Protein Binding in Human Brain With the Novel Radioligand [18F]‐FEPPA and Positron Emission Tomography,” Journal of Cerebral Blood Flow and Metabolism 31, no. 8 (2011): 1807–1816, 10.1038/jcbfm.2011.55. 21522163 PMC3170950

[24] S. Hafizi , H. H. Tseng , N. Rao , et al., “Imaging Microglial Activation in Untreated First‐Episode Psychosis: A PET Study With [(18)F]FEPPA,” American Journal of Psychiatry 174, no. 2 (2017): 118–124, 10.1176/appi.ajp.2016.16020171. 27609240 PMC5342628

[25] S. Shakory , J. J. Watts , S. Hafizi , et al., “Hippocampal Glutamate Metabolites and Glial Activation in Clinical High Risk and First Episode Psychosis,” Neuropsychopharmacology 43 (2018): 2249–2255, 10.1038/s41386-018-0163-0. 30087434 PMC6135774

[26] C. Ghadery , Y. Koshimori , S. Coakeley , et al., “Microglial Activation in Parkinson's Disease Using [(18)F]‐FEPPA,” Journal of Neuroinflammation 14, no. 1 (2017): 8, 10.1186/s12974-016-0778-1. 28086916 PMC5234135

[27] Y. Koshimori , J. H. Ko , R. Mizrahi , et al., “Imaging Striatal Microglial Activation in Patients With Parkinson's Disease,” PLoS ONE 10, no. 9 (2015): e0138721, 10.1371/journal.pone.0138721. 26381267 PMC4575151

[28] E. Setiawan , S. Attwells , A. A. Wilson , et al., “Association of Translocator Protein Total Distribution Volume With Duration of Untreated Major Depressive Disorder: A Cross‐Sectional Study,” Lancet Psychiatry 5, no. 4 (2018): 339–347, 10.1016/S2215-0366(18)30048-8. 29496589

[29] H. Li , A. P. Sagar , and S. Keri , “Translocator Protein (18kDa TSPO) Binding, a Marker of Microglia, Is Reduced in Major Depression During Cognitive‐Behavioral Therapy,” Progress in Neuro‐Psychopharmacology & Biological Psychiatry 83 (2018): 1–7, 10.1016/j.pnpbp.2017.12.011. 29269262

[30] D. Knezevic , N. P. L. Verhoeff , S. Hafizi , et al., “Imaging Microglial Activation and Amyloid Burden in Amnestic Mild Cognitive Impairment,” Journal of Cerebral Blood Flow and Metabolism 38 (2017): 1895, 10.1177/0271678X17741395. PMC6259323 29135331

[31] I. Suridjan , B. G. Pollock , N. P. Verhoeff , et al., “In‐Vivo Imaging of Grey and White Matter Neuroinflammation in Alzheimer's Disease: A Positron Emission Tomography Study With a Novel Radioligand, [18F]‐FEPPA,” Molecular Psychiatry 20, no. 12 (2015): 1579–1587, 10.1038/mp.2015.1. 25707397 PMC8026116

[32] A. R. Soares and M. R. Picciotto , “Nicotinic Regulation of Microglia: Potential Contributions to Addiction,” Journal of Neural Transmission (Vienna) 131, no. 5 (2024): 425–435, 10.1007/s00702-023-02703-9. PMC11189589 37778006

[33] W. Zhang , H. Lin , M. Zou , et al., “Nicotine in Inflammatory Diseases: Anti‐Inflammatory and Pro‐Inflammatory Effects,” Frontiers in Immunology 13 (2022): 826889, 10.3389/fimmu.2022.826889. 35251010 PMC8895249

[34] L. Chang , H. Liang , S. R. Kandel , and J. J. He , “Independent and Combined Effects of Nicotine or Chronic Tobacco Smoking and HIV on the Brain: A Review of Preclinical and Clinical Studies,” Journal of Neuroimmune Pharmacology 15, no. 4 (2020): 658–693, 10.1007/s11481-020-09963-2. 33108618

[35] A. Khanna , M. Guo , M. Mehra , and W. Royal , “Inflammation and Oxidative Stress Induced by Cigarette Smoke in Lewis Rat Brains,” Journal of Neuroimmunology 254, no. 1–2 (2013): 69–75, 10.1016/j.jneuroim.2012.09.006. 23031832 PMC3534934

[36] W. Royal, III , A. Can , T. D. Gould , et al., “Cigarette Smoke and Nicotine Effects on Brain Proinflammatory Responses and Behavioral and Motor Function in HIV‐1 Transgenic Rats,” Journal of Neurovirology 24, no. 2 (2018): 246–253, 10.1007/s13365-018-0623-7. 29644536 PMC5940844

[37] A. L. Brody , R. Hubert , R. Enoki , et al., “Effect of Cigarette Smoking on a Marker for Neuroinflammation: A [(11)C]DAA1106 Positron Emission Tomography Study,” Neuropsychopharmacology 42, no. 8 (2017): 1630–1639, 10.1038/npp.2017.48. 28262740 PMC5518907

[38] A. L. Brody , M. A. Mandelkern , E. D. London , et al., “Cigarette Smoking Saturates Brain alpha4beta2 Nicotinic Acetylcholine Receptors,” ArchGenPsychiatry 63, no. 8 (2006): 907–915. 10.1001/archpsyc.63.8.907 PMC2773659 16894067

[39] A. L. Brody , M. A. Mandelkern , M. R. Costello , et al., “Brain Nicotinic Acetylcholine Receptor Occupancy: Effect of Smoking a Denicotinized Cigarette,” International Journal of Neuropsychopharmacology 12, no. 3 (2009): 305–316. 18706128 10.1017/S146114570800922X PMC2773668

[40] A. L. Brody , M. A. Mandelkern , E. D. London , et al., “Effect of Secondhand Smoke on Occupancy of Nicotinic Acetylcholine Receptors in Brain,” Archives of General Psychiatry 68, no. 9 (2011): 953–960, 10.1001/archgenpsychiatry.2011.51. 21536968 PMC3380615

[41] A. L. Brody , A. G. Mukhin , J. La Charite , et al., “Up‐Regulation of Nicotinic Acetylcholine Receptors in Menthol Cigarette Smokers,” International Journal of Neuropsychopharmacology 16, no. 5 (2013): 957–966, 10.1017/S1461145712001022. 23171716 PMC3758251

[42] K. P. Cosgrove , J. Batis , F. Bois , et al., “beta2‐Nicotinic Acetylcholine Receptor Availability During Acute and Prolonged Abstinence From Tobacco Smoking,” Archives of General Psychiatry 66, no. 6 (2009): 666–676, 10.1001/archgenpsychiatry.2009.41. 19487632 PMC2796827

[43] A. L. Brody , D. Gehlbach , L. Y. Garcia , et al., “Effect of Overnight Smoking Abstinence on a Marker for Microglial Activation: A [11(C)]DAA1106 Positron Emission Tomography Study,” Psychopharmacology 235, no. 12 (2018): 3525–3534. 30343364 10.1007/s00213-018-5077-3 PMC6497451

[44] P. M. Lai , M. Araga , A. Hamilton , and M. Armogida , “Pharmacokinetic Characterization of a Prototype Mini Nicotine Lozenge,” Advances in Therapy 38, no. 7 (2021): 3997–4012, 10.1007/s12325-021-01798-4. 34105089

[45] M. J. Jarvis , M. A. Russell , N. L. Benowitz , and C. Feyerabend , “Elimination of Cotinine From Body Fluids: Implications for Noninvasive Measurement of Tobacco Smoke Exposure,” American Journal of Public Health 78, no. 6 (1988): 696–698, 10.2105/ajph.78.6.696. 3369603 PMC1350287

[46] N. L. Benowitz and P. Jacob, 3rd , “Metabolism of Nicotine to Cotinine Studied by a Dual Stable Isotope Method,” Clinical Pharmacology & Therapeutics 56, no. 5 (1994): 483–493. 7955812 10.1038/clpt.1994.169

[47] S. Feng , S. Kapur , M. Sarkar , et al., “Respiratory Retention of Nicotine and Urinary Excretion of Nicotine and Its Five Major Metabolites in Adult Male Smokers,” Toxicology Letters 173, no. 2 (2007): 101–106, 10.1016/j.toxlet.2007.06.016. 17716838

[48] J. H. Meyer , S. Cervenka , M. J. Kim , W. C. Kreisl , I. D. Henter , and R. B. Innis , “Neuroinflammation in Psychiatric Disorders: PET Imaging and Promising new Targets,” Lancet Psychiatry 7, no. 12 (2020): 1064–1074, 10.1016/S2215-0366(20)30255-8. 33098761 PMC7893630

[49] American Psychiatric Association , Diagnostic and Statistical Manual of Mental Disorders: DSM‐5 (American Psychiatric Association, 2013).

[50] T. F. Heatherton , L. T. Kozlowski , R. C. Frecker , and K. O. Fagerström , “The Fagerström Test for Nicotine Dependence: A Revision of the Fagerström Tolerance Questionnaire,” British Journal of Addiction 86 (1991): 1119–1127. 1932883 10.1111/j.1360-0443.1991.tb01879.x

[51] K. O. Fagerström , “Measuring the Degree of Physical Dependence to Tobacco Smoking With Reference to Individualization of Treatment,” Addictive Behaviors 3 (1978): 235–241. 735910 10.1016/0306-4603(78)90024-2

[52] K. K. Yoder , K. Nho , S. L. Risacher , S. Kim , L. Shen , and A. J. Saykin , “Influence of TSPO Genotype on 11C‐PBR28 Standardized Uptake Values,” Journal of Nuclear Medicine 54, no. 8 (2013): 1320–1322, 10.2967/jnumed.112.118885. 23785173 PMC3740346

[53] D. M. McNair , D. Lorr , and L. F. Droppelman , Manual for the Profile of Mood States (Educational Testing Services, 1988).

[54] “Key Substance Use and Mental Health Indicators in the United States: Results From the 2019 National Survey on Drug Use and Health,” (2020).

[55] R. D. Goodwin , L. R. Pacek , J. Copeland , et al., “Trends in Daily Cannabis Use Among Cigarette Smokers: United States, 2002‐2014,” American Journal of Public Health 108, no. 1 (2018): 137–142, 10.2105/AJPH.2017.304050. 29161058 PMC5719676

[56] K. E. Moeller , J. C. Kissack , R. S. Atayee , and K. C. Lee , “Clinical Interpretation of Urine Drug Tests: What Clinicians Need to Know About Urine Drug Screens,” Mayo Clinic Proceedings 92, no. 5 (2017): 774–796, 10.1016/j.mayocp.2016.12.007. 28325505

[57] A. A. Wilson , A. Garcia , J. Parkes , et al., “Radiosynthesis and Initial Evaluation of [18F]‐FEPPA for PET Imaging of Peripheral Benzodiazepine Receptors,” Nuclear Medicine and Biology 35, no. 3 (2008): 305–314, 10.1016/j.nucmedbio.2007.12.009. 18355686

[58] N. Vasdev , D. E. Green , D. C. Vines , et al., “Positron‐Emission Tomography Imaging of the TSPO With [(18)F]FEPPA in a Preclinical Breast Cancer Model,” Cancer Biotherapy & Radiopharmaceuticals 28, no. 3 (2013): 254–259, 10.1089/cbr.2012.1196. 23350894

[59] A. L. Brody , M. A. Mandelkern , E. D. London , et al., “Brain Metabolic Changes During Cigarette Craving,” Archives of General Psychiatry 59 (2002): 1162–1172. 12470133 10.1001/archpsyc.59.12.1162

[60] A. L. Brody , R. E. Olmstead , E. D. London , et al., “Smoking‐Induced Ventral Striatum Dopamine Release,” American Journal of Psychiatry 161, no. 7 (2004): 1211–1218. 15229053 10.1176/appi.ajp.161.7.1211

[61] A. L. Brody , M. A. Mandelkern , R. E. Olmstead , et al., “Gene Variants of Brain Dopamine Pathways and Smoking‐Induced Dopamine Release in the Ventral Caudate/Nucleus Accumbens,” Archives of General Psychiatry 63, no. 7 (2006): 808–816. 16818870 10.1001/archpsyc.63.7.808 PMC2873693

[62] A. L. Brody , M. A. Mandelkern , R. E. Olmstead , et al., “Ventral Striatal Dopamine Release in Response to Smoking a Regular vs a Denicotinized Cigarette,” Neuropsychopharmacology 34, no. 2 (2009): 282–289. 18563061 10.1038/npp.2008.87 PMC2777990

[63] F. Yasuno , J. Kosaka , M. Ota , et al., “Increased Binding of Peripheral Benzodiazepine Receptor in Mild Cognitive Impairment‐Dementia Converters Measured by Positron Emission Tomography With [(11)C]DAA1106,” Psychiatry Research 203, no. 1 (2012): 67–74, 10.1016/j.pscychresns.2011.08.013. 22892349

[64] A. Takano , R. Arakawa , H. Ito , et al., “Peripheral Benzodiazepine Receptors in Patients With Chronic Schizophrenia: A PET Study With [11C]DAA1106,” International Journal of Neuropsychopharmacology 13, no. 7 (2010): 943–950, 10.1017/S1461145710000313. 20350336

[65] M. D. Walker , K. Dinelle , R. Kornelsen , et al., “[11C]PBR28 PET Imaging Is Sensitive to Neuroinflammation in the Aged Rat,” Journal of Cerebral Blood Flow and Metabolism 35, no. 8 (2015): 1331–1338, 10.1038/jcbfm.2015.54. 25833342 PMC4528008

[66] M. Toth , J. Doorduin , J. Haggkvist , et al., “Positron Emission Tomography Studies With [11C]PBR28 in the Healthy Rodent Brain: Validating SUV as an Outcome Measure of Neuroinflammation,” PLoS ONE 10, no. 5 (2015): e0125917, 10.1371/journal.pone.0125917. 25996996 PMC4440816

[67] A. T. Hillmer , C. M. Sandiego , J. Hannestad , et al., “In Vivo Imaging of Translocator Protein, a Marker of Activated Microglia, in Alcohol Dependence,” Molecular Psychiatry 22, no. 12 (2017): 1759–1766, 10.1038/mp.2017.10. 28242869 PMC5573660

[68] S. W. Kim , C. E. Wiers , R. Tyler , et al., “Influence of Alcoholism and Cholesterol on TSPO Binding in Brain: PET [(11)C]PBR28 Studies in Humans and Rodents,” Neuropsychopharmacology 43, no. 9 (2018): 1832–1839, 10.1038/s41386-018-0085-x. 29777199 PMC6046047

[69] P. F. Engstrom , M. L. Clapper , and R. A. Schnoll , “Physiochemical Composition of Tobacco Smoke,” in Holland‐Frei Cancer Medicine, 6th ed., (BC Decker Inc, 2003).

[70] “Program NT. Tobacco‐Related Exposures. Report on Carcinogens Fourteenth Edition,” U.S. Department of Health and Human Services, Public Health Service, National Toxicology Program , (2016).

[71] K. T. Pan , G. S. Leonardi , M. Ucci , and B. Croxford , “Can Exhaled Carbon Monoxide Be Used as a Marker of Exposure? A Cross‐Sectional Study in Young Adults,” International Journal of Environmental Research and Public Health 18, no. 22 (2021): 11893, 10.3390/ijerph182211893. 34831647 PMC8617968

[72] W. K. Jo and K. W. Pack , “Utilization of Breath Analysis for Exposure Estimates of Benzene Associated With Active Smoking,” Environmental Research 83, no. 2 (2000): 180–187, 10.1006/enrs.2000.4059. 10856191

[73] S. Chelland Campbell , R. J. Moffatt , and B. A. Stamford , “Smoking and Smoking Cessation—The Relationship Between Cardiovascular Disease and Lipoprotein Metabolism: A Review,” Atherosclerosis 201, no. 2 (2008): 225–235, 10.1016/j.atherosclerosis.2008.04.046. 18565528

[74] Y. H. Fan Chiang , Y. W. Lee , F. Lam , C. C. Liao , C. C. Chang , and C. S. Lin , “Smoking Increases the Risk of Postoperative Wound Complications: A Propensity Score‐Matched Cohort Study,” International Wound Journal 20, no. 2 (2023): 391–402, 10.1111/iwj.13887. 35808947 PMC9885463

[75] K. Lasser , J. W. Boyd , S. Woolhandler , D. U. Himmelstein , D. McCormick , and D. H. Bor , “Smoking and Mental Illness—A Population‐Based Prevalence Study,” Journal of the American Medical Association 284, no. 20 (2000): 2606–2610. 11086367 10.1001/jama.284.20.2606

[76] UK NHS , https://www.nhs.uk/better‐health/quit‐smoking/, (2024).

[77] D. Gritti , G. Delvecchio , A. Ferro , C. Bressi , and P. Brambilla , “Neuroinflammation in Major Depressive Disorder: A Review of PET Imaging Studies Examining the 18‐kDa Translocator Protein,” Journal of Affective Disorders 292 (2021): 642–651, 10.1016/j.jad.2021.06.001. 34153835

[78] E. Setiawan , A. A. Wilson , R. Mizrahi , et al., “Role of Translocator Protein Density, a Marker of Neuroinflammation, in the Brain During Major Depressive Episodes,” JAMA Psychiatry 72, no. 3 (2015): 268–275, 10.1001/jamapsychiatry.2014.2427. 25629589 PMC4836849

[79] Y. Li and S. S. Hecht , “Carcinogenic Components of Tobacco and Tobacco Smoke: A 2022 Update,” Food and Chemical Toxicology 165 (2022): 113179, 10.1016/j.fct.2022.113179. 35643228 PMC9616535

[80] A. Rodgman and T. Perfetti , The Chemical Components of Tobacco and Tobacco Smoke, 2nd ed., (CRC Press, 2013).

[81] A. R. Peralta and V. P. Guntur , “Safety and Efficacy of Electronic Cigarettes: A Review,” Missouri Medicine 111, no. 3 (2014): 238–244. 25011347 PMC6179561

[82] D. E. Schraufnagel , F. Blasi , M. B. Drummond , et al., “Electronic Cigarettes: A Position Statement of the Forum of International Respiratory Societies,” American Journal of Respiratory and Critical Care Medicine 190, no. 6 (2014): 611–618, 10.1164/rccm.201407-1198PP. 25006874

[83] P. Caponnetto , A. Alamo , M. Maglia , D. Saitta , F. Benfatto , and R. Polosa , “Electronic Cigarette: Current Overview and Future Perspectives,” Epidemiologia e Prevenzione 38, no. 2 (2014): 138–141 Sigaretta elettronica: panoramica attuale e prospettive future. 24986413

[84] I. T. Agaku , B. A. King , C. G. Husten , et al., “Tobacco Product Use Among Adults—United States, 2012–2013,” MMWR. Morbidity and Mortality Weekly Report 63, no. 25 (2014): 542–547. 24964880 PMC5779380

[85] J. W. Young , C. V. Barback , L. A. Stolz , et al., “MicroPET Evidence for a Hypersensitive Neuroinflammatory Profile of gp120 Mouse Model of HIV,” Psychiatry Research: Neuroimaging 321 (2022): 111445, 10.1016/j.pscychresns.2022.111445. 35101828

[86] E. Nutma , N. Fancy , M. Weinert , et al., “Translocator Protein Is a Marker of Activated Microglia in Rodent Models But Not Human Neurodegenerative Diseases,” Nature Communications 14, no. 1 (2023): 5247, 10.1038/s41467-023-40937-z. PMC10462763 37640701

[87] G. Rizzo , M. Veronese , M. Tonietto , P. Zanotti‐Fregonara , F. E. Turkheimer , and A. Bertoldo , “Kinetic Modeling Without Accounting for the Vascular Component Impairs the Quantification of [(11)C]PBR28 Brain PET Data,” Journal of Cerebral Blood Flow and Metabolism 34, no. 6 (2014): 1060–1069, 10.1038/jcbfm.2014.55. 24667911 PMC4050251

[88] F. E. Turkheimer , G. Rizzo , P. S. Bloomfield , et al., “The Methodology of TSPO Imaging With Positron Emission Tomography,” Biochemical Society Transactions 43, no. 4 (2015): 586–592, 10.1042/BST20150058. 26551697 PMC4613512

